# Erythrocyte DHA and AA in infancy is not associated with developmental status and cognitive functioning five years later in Nepalese children

**DOI:** 10.1186/s12937-018-0375-5

**Published:** 2018-07-19

**Authors:** Sigrun Henjum, Ingrid Kvestad, Merina Shrestha, Manjeswori Ulak, Ram K. Chandyo, Andrew L. Thorne-Lyman, Prakash S. Shrestha, Marian Kjellevold, Mari Hysing, Tor A. Strand

**Affiliations:** 1 OsloMet – Oslo Metropolitan University, Postboks 4, St. Olavs plass, Oslo, Norway; 2 Regional Center for Child and Youth Mental Health and Child Welfare, West, Uni Research Health, Bergen, Norway; 3 Department of Child Health, Tribhuvan University Teaching Hospital, Kathmandu, Nepal; 4 Department of Community Medicine, Kathmandu Medical College, P.O. Box 21266, Kathmandu, Nepal; 5 Center for Human Nutrition, Department of International Health, Johns Hopkins Bloomberg School of Public Health, Baltimore, MD USA; 6 Harvard T.H. Chan School of Public Health, Boston, MA USA; 7 Institute of Marine Research, 5817 Bergen, Norway; 8 Division for Medical Services, Innlandet Hospital Trust, Lillehammer, Norway; 9 The Center for International Health, University of Bergen, Bergen, Norway

**Keywords:** Polyunsaturated fatty acids, Plasma phospholipids, DHA, AA, Developmental status, Cognitive functioning, Follow up study, Nepalese children

## Abstract

**Background:**

Long chain polyunsaturated fatty acids (LCPUFA) especially docosahexaenoic acid (DHA) and arachidonic acid (AA) are crucial for normal brain development in utero and in early infancy. Data on fatty acid status and cognitive development in infants and children from low-income countries are scarce.

**Methods:**

We examined the association between the DHA and AA status in infancy (*n* = 320) and developmental status and cognitive functioning five years later. At five years of age, we measured development by the Ages and Stages Questionnaire 3rd. ed. (ASQ-3) and cognitive functioning by subtests from the neuropsychological test battery NEPSY II. In addition, infant fatty acid composition in red blood cells (RBC) was analyzed. In multiple linear and logistic regression models, we estimated the associations between DHA and AA status in infancy and scores on the ASQ-3 and the NEPSY II subtests.

**Results:**

There were no notable associations between infant AA and DHA status, and the scores on the ASQ-3 and the NEPSY II subtests five years later. It should be noted that we found better than expected concentrations of erythrocyte DHA and AA among the infants, and the ASQ scores were left-skewed, which limited the ability to identify associations.

**Conclusion:**

DHA and AA status in infancy is seemingly not related to neurodevelopment measured 5 years later in this peri-urban population from Nepal.

## Background

Adequate nutrition, especially during pregnancy and infancy, is necessary for normal brain development [1, 2]. Long chain polyunsaturated fatty acids (LCPUFA) such as docosahexaenoic acid (DHA) and arachidonic acid (AA) are crucial in this process [2–5]. DHA and AA represent over 30% of all fatty acids in the gray matter of the human brain and the highest concentrations are found in the synapses of the neurons, where they have an important role in signal transduction [6]. DHA regulates numerous neuronal and glial cell processes including neurogenesis, neuroplasticity, neurite outgrowth, synaptogenesis and membrane fluidity, which in turn supports membrane protein functions affecting the speed of signal transduction and neurotransmission [7–10]. DHA and AA influence neurotransmission as precursors in the eicosanoid metabolism, and are important for normal function of the brain [11]. AA and DHA can either be supplied by dietary intake or synthesized from their essential precursors linoleic acid (LA) or a-linolenic acid (ALA), respectively. The conversion of ALA to eicosapentaenoic acid (precursor of DHA) is assumed to be low, and this may explain why formula-fed infants not consuming DHA have lower levels of DHA in brain compared to breast-fed infants [12]. Postpartum levels of DHA in the mother have been shown to be determined by DHA levels in pregnancy [13]. Thus, both insufficient maternal fatty acid status in pregnancy and the infants dietary sources of DHA after birth can put the infant at risk for deficiency in a critical period for the development of the central nervous system (CNS) [2, 12].

In the scientific literature, the role of LCPUFA on cognitive development is not conclusive. There is some evidence that LCPUFA supplementation has an effect on cognition among preterm [1, 3, 14–16] and low birth weight (LBW) infants [3, 4], in those who are LCPUFA deficient, [17, 18] or at risk for deficiency [6]. In full-term infants, however, beneficial effects of LCPUFA on cognition have been found in some [16, 18–20], but not in all studies [3, 6, 14, 21, 22]. The lack of an association of LCPUFA on cognition may have several explanations including differences in DHA and AA concentration of supplements/formula, duration of intervention, the type of development assessments, age at assessment, and the possibility of effect modification by unmeasured genetic polymorphisms (FADS) [23]. Emerging data demonstrate that the presence of FADS polymorphisms among study populations introduces substantial variation not recognized in initial studies in this field [23]. Few studies conducted in low-income countries have examined associations between fatty-acid status in infants and their longer-term development [1, 3, 24, 25].

In Bhaktapur, Nepal, despite low dietary intake of LA, ALA and LCPUFA, many infants and women had better DHA and AA status than expected [26, 27]. We observed a wide range of DHA and AA status, potentially making this a useful population to study associations with outcomes, such as child development and cognitive functioning. The aim of the present study is therefore to examine the association between the AA and DHA status in infancy and developmental status and cognitive functioning 5 years later in a representative sample of children from Nepal. In a recently published paper from the same study, we demonstrated that vitamin B12 status was significantly associated with these cognitive outcomes [28].

## Methods

### Design, recruitment, and participants

**Fig. 1:**
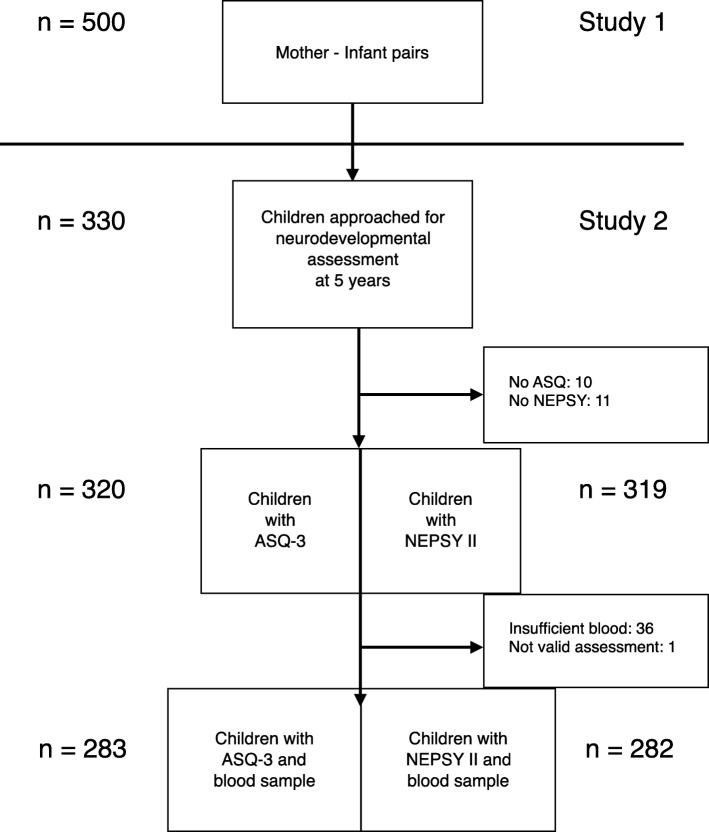
Flowchart of the study participants

We used a two-stage cluster sampling procedure whereby 66 neighborhoods (“toles”) were randomly selected as the primary sampling unit from 160. We listed all women living in these toles, and randomly selected women and infant pairs. The inclusion criteria for the study were that both mothers and children had no on-going clinically assessed infections that the infants were between 2 and 11 months, resided in the selected clusters, were willing to provide their household information, and consented to participate. Details of the selection criteria and a flow chart of the recruitment of the study subjects have been published elsewhere [29]. The study obtained ethical clearance from the institutional review board at the Institute of Medicine in Kathmandu, Nepal and the Regional Committee for Medical and Health Research Ethics in Norway. In 2012 and 2013, approximately five years after the first inclusions, we approached 330 of the children from the initial cohort of 500 women-child pairs for inclusion to developmental and neuropsychological assessment (Study 2) (Fig. 1). Of these 330, 10 children did not perform the Ages and Stages Questionnaire 3rd edition (ASQ-3) and 11 did not performed the NEPSY II. One child was excluded from the analysis due to the suspicion of a medical condition that undermined the validity of the assessment of that specific child, and the final number of valid assessments was 320 for ASQ-3 and 319 for the NEPSY II. In 36 children, we were not able to obtain sufficient blood at baseline, and thus the number of children in the regression analysis is 282 and 283. There were no major differences between the current subsample and the original sample in the demographic features such as child characteristics, child nutritional status and family situation at baseline, results are presented elsewhere [28]. Written consent was obtained from mothers at baseline and for the follow-up visits.

### Laboratory procedures

A trained phlebotomist drew approximately 3 mL of whole blood from the cubital vein at enrolment when the infant were between 2 and 11 months, into olypropylene tubes with lithium heparin (Sarstedt, Germany). The samples were then centrifuged (760×g, for 10 min, room temperature) and plasma was allocated into polypropylene vials (Eppendorf, Hinz, Germany). Samples were stored at − 20 °C at the field site laboratory until they were transported with an ice pack to the central laboratory in Kathmandu at the end of each day. There, samples were stored at − 80 °C until transport on dry ice to Norway. The fatty acid composition of total RBC was determined by ultrafast gas chromatography (UFGC) (Thermo Electron Corporation, Massachusetts, USA), using a method developed by Araujo P, Nguyen TT, Froyland L, Wang J and Kang JX [30]. Briefly, 50 μl homogenized samples were mixed with boron trifluoride (BF3) and internal standard (19:0 methyl ester), followed by extraction with hexane. The fatty acid composition was calculated using a labdataprogram (Chromeleon 6.80, Dionex Corporation, California, USA), connected to the UFGC and identification ascertained by standard mixtures of methyl esters (Nu-Chek, Minnesota, USA). Limit of quantification was 10 μg fatty acid/g samples (wet weight. *w*/w). The certified reference materials (CRM) CRM 162 (soy oil) and CRM 163 (pig fat) controlled the analytical quality of the method and systematic errors. The fatty acid composition of cooking oils was analyzed by gas liquid chromatography (GLC, Trace GC 2000, Termo) according to previously described method [31]. Total lipid content was extracted, filtered, evaporated, saponified and fatty acids were esterified. The methyl esters were separated using Auto-GC (Instrument-Teknikk AS, Norway), equipped with a 50 m CP sil 88 (Chrompack) fused silica capillary column (id:0.32 mm), using “cold on column” injection, temperature program (60 °C (25 °C/min) to 160 (25 °C/min) to 190 (25 °C/min) to 220) and flame ionization detector. The fatty acid composition was calculated using a labdataprogram (Turbochrom Navigator, Version 6.1), connected to the GLC and identification as ascertained by standard mixtures of methyl esters (Nu-Chek, Elyian, USA). Nonadecanoic acid (19:0) methyl ester was used as internal standard. Limit of quantification (LOQ) was 10 μg fatty acid/g sample (wet weight, *w*/w).

### Cognitive assessments

We used the Ages and Stages Questionnaire 3rd. edition and subtests from the NEPSY II, to assess neurodevelopment and cognitive functioning in the present study. A local pediatrician and a psychologist, both experienced in child development assessment, were trained to perform the assessments by two Norwegian clinical psychologists. The local psychologist performed most of the assessments under close supervision. Assessments were conducted at the study clinic in a well-lit room free from distractions. The children were followed to the clinic by their caregivers, and the caregivers had the opportunity to sit in the back of the room during the assessments. The sessions lasted for approximately one hour. A detailed description of the translation, training and assessment procedures are presented elsewhere [28].

The ASQ-3 is a comprehensive developmental screening, standardized for children 1–66 months with age-appropriate questionnaires [32]. The questionnaires contain 30 items that sums up to five subscales: communication, gross motor, fine motor, problem solving and personal-social (possible scores range from 0 to 60), and a total score (possible score range from 0 to 300). The ASQ-3 is designed to be answered by caregivers, but can also be used for direct assessment of the child by a trained professional [33] as we did in the current study. The examiner (the trained psychologist) aimed to answer the items in the questionnaire during the sessions and used a collection of standardized material (e.g. large and small balls, pen, paper and scissors) in the assessments. If the examiner could not answer the items through direct observation during the sessions, the response relied on answers from the caregiver. The 60 months questionnaire (age range 57–66 months) was used for the present study. In total, 160 of the 320 participants were above 66 months at the time of the assessment; however, we decided to perform the ASQ-3 for all children in the sample. The mean (SD) age in months at testing was 66.7 (3.4). The questionnaire was translated and back translated and culturally adapted particularly for this Nepalese setting according to standard procedures. A detailed description of the translation, training and assessment procedures are presented elsewhere [28].

The NEPSY-II is a comprehensive neuropsychological test battery that consists of 32 subtests in six functional domains for children aged 3 to 16 years [34]. The battery is flexible and allows for individual administration. The following six age–appropriate subtests were administrated in the present study: Inhibition, Statue, Visuo-motor Precision, Affect Recognition, Geometric Puzzles and Block construction. Since norms are not available for the Nepalese population, we calculated the scaled scores based on the American norms [34]. The NEPSY II scaled scores have a total possible range from 1 to 19, with 10 (3) as the mean (SD).

### Anthropometric measurements

Children were weighed on a UNICEF electronic pediatric scale (SECA, Germany) and length was measured using a locally made wooden board with an accuracy of ±0.1 cm. The Z-scores for height-for-age (HAZ) and weight-for-age (WAZ) were calculated using WHO growth reference standard from 2006 [35]. Children were classified as stunted or underweight if their HAZ or WAZ was <− 2 SD, respectively.

### Data management and statistics

Data were analysed using STATA version 14 (StataCorp., College Station,TX, USA). Data were expressed both as means (±SD) and median (IQR). We used multiple linear and logistic regression models to estimate the association between fatty acid status in infancy and the scores on ASQ-3 and the NEPSY II subtests approximately 5 years later. DHA and AA concentrations were used as the exposure variables in the different regression models both as linear variables and categorized in tertiles. The ASQ-3 subscales were highly skewed, and we dichotomized the scores at the 25th percentile and used logistic regression when analysing the subscales. An OR more than one indicates an increased risk of being in the lowest 25th percentile for each increment of a biomarker unit. We also undertook the multiple regression analyses adjusting for variables listed in Table 1, only variables that changed the exposure-outcome relationships by more than 15% were included in the final models. The variables included in the multiple regression models are sex, age at baseline, weight-for-age-z-score and age at testing. We present only the analyses of the linear exposure variables, and present both crude and adjusted estimates. The effect estimates are expressed as linear regression coefficient and odds ratios (OR), and a *p*-value-threshold of 0.05 was used to denote statistical significance. Post hoc, we stratified the analyses on children with birthweight less than 2500 g and children 2500 g and above.

**Table 1:** Variables assessed in the multiple regression models to measure the association between fatty acids (AA and DHA) and the ASQ-3 and NEPSY II scores

	Continuous	Categorical
Sex		male or female
Age at enrollment	months	
Exclusive breastfeeding at enrollment	–	yes or no
Iron status at enrollment	μmol/L	–
Height-for-age z-scores at enrollment	z scores	
Weight-for-height z-scores at enrollment	z scores	
Weight-for-age z-scores at enrollment	z scores	
Parity		1–2 or 2 and more
Energy intake mother at enrollment	Kcal /day	
Folate supplementation in pregnancy		yes or no
Living in joint family		yes or no
Family owns land		yes or no
Number of rooms in the home	number	–
Mothers age	years	–
Level of education, mother		less than 10th grade or 10th grade and more
Level of education, father		less than 10th grade or 10th grade and more
Occupation, mother		no work/agricultural or other work
Occupation, father		no work/agricultural or other work

## Results

Child, household and maternal characteristics and family situation at baseline are presented in Table 2. The mean (SD) age of the children at baseline was 7.0 (2.9) months and birthweight was 2872 (476) grams. Fourteen percent of the children had a birthweight below 2500 g. In total, 5% of the children were classified as underweight and 9% as stunted. Around half of the mothers and 70% of the fathers had more than 10th grade.

**Table 2:** Child, parent, and household characteristics at baseline (n = 320)

Characteristics	% (n)
Boys	55.9 (179)
Age infant, months^a^	7.0 (2.9)
Birth weight^a^	2872 (476)
Birth weight < 2500 g	14 (45)
Exclusively breast at enrolment	13.6 (43)
Underweight (<−2 z score weight for age)	5 (16)
Stunted (<−2 z score length for age)	9 (27)
Mother:	
Age mother^a^	26.1 (4.2)
Less than grade 10 of schooling	48.2 (147)
10th grade and more	51.8 (158)
Working outside home	26.2 (80)
Father:	
Less than grade 10	30.3 (94)
10th grade and more	69.7 (216)
Working outside home	93.1 (281)
Household:	
Joint family	53.8 (170)
Own land	57.6 (182)

^a^Mean (SD)

Characteristics of the total sample is presented elsewhere (24)

Median (IQR) and mean (range) for the total ASQ-3 score and the five subscales are presented in Table 3. The median (IQR) for the total score was 270 (255–285). Mean (SD) for the total raw and corresponding scaled NEPSY II scores in the 319 children are shown in Table 4. The mean scaled scores range from 5.9 (2.8) to 12.5 (1.7), with the lowest scores in the Visuo-motor precision total completion time subtest, and the highest in the Statue total score subtest.

**Table 3:** ASQ-3 total and subscale scores in 320 Nepali children

Characteristics	Median (IQR)	Mean (Range)
Total ASQ-3	270 (255–285)	265.8 (45–300)
Subscales:		
Communication	50 (40–55)	47.9 (0–60)
Gross motor	60 (60–60)	57.6 (20–60)
Fine motor	50 (45–55)	49.1 (0–60)
Problem solving	60 (55–60)	55.9 (0–60)
Personal Social	60 (50–60)	55.1 (15–60)

Total possible range is 0–300 for total score and 0–60 for the subscale score

**Table 4:** NEPSY-II raw and scaled scores in 319 Nepali Children

		Raw scores (SD)	Mean scaled scores (SD)^a^	Range scaled scores
Attention and Executive Functioning				
1	Inhibition-Naming Completion Time Total	95.0 (23.9)	10.2 (2.9)	1–19
	Inhibition-Inhibition Completion Time Total	138.8 (38.6)	9.8 (2.8)	1–19
	Inhibition Total Errors	20.8 (16.3)	8.7 (4.3)	1–19
2	Statue Total Score	27.9 (2.8)	12.5 (1.7)	5–14
Sensorimotor				
3	Visuo-motor Precision Total Completion Time	184.3 (56.3)	5.9 (2.8)	1–19
	Visuo-motor Precision Combined Scaled Score		10.3 (2.2)	4–19
Social Perception				
4	Affect Recognition Total Score	14.5 (3.3)	7.6 (3.3)	1–15
Visuospatial Processing				
5	Block Construction Total Score	7.4 (1.7)	7.9 (2.5)	1–15
6	Geometric Puzzles Total Score^b^	13.3 (3.0)		

^a^The NEPSY II scaled scores are calculated based on US norm. The scaled scores have a total possible range from 1 to 19, with 10(3) as mean (SD)

^b^Scaled scores are not available for this age range

Fatty acid composition of red blood cells in infants is presented in Table 5. The mean concentrations of DHA and AA for all infants were 116 and 311 μg/g, respectively. DHA was the main n-3 fatty acid, as AA was the major n-6 fatty acid.

**Table 5:** Fatty acid composition of red blood in infants (*n* = 303)^a^

	Infants (n = 303)	
	(μg/ml)	%
Total SAFA	948 (99)	41
Total MUFA	449 (76)	19
Total PUFA	906 (158)	38
Total *n*-6 PUFA	685 (128)	29
Total *n*-3 PUFA	222 (44)	9.4
18:2 *n*-6 (LA)	269 (76)	11.3
18:3 *n*-3 (ALA)	5.4 (5)	0.2
20:4 *n*-6 (AA)	311 (59)	13.2
20:5 *n*-3 (EPA)	8.3 (4)	0.3
22:6 *n*-3 (DHA)	116 (22)	4.9
22:5 *n-3* (DPA)	37 (11)	1.6
n-6/n-3 PUFA *Ratio*	3.2 (0.6)	
Sum fatty acids	2345 (293)	
Omega 3 index %^b^		5.2

Mean ± SD

^a^Has been presented elsewhere (21)

^b^Sum %EPA + %DHA (% of sum of total fatty acids)

Associations between DHA and AA and the total and subscale scores of the ASQ-3 in the Nepalese preschoolers are presented in Table 6. In both the multiple adjusted linear and logistic regression analysis we found no associations between ASQ-3 total and subscale scores with either AA or DHA. None of our analytical approaches (i.e. exposures categorized on tertiles) revealed any significant associations between developmental scores and fatty acid status. Low birth weight did not modify any of the associations (data not shown).

**Table 6:** Associations between DHA and AA and the total and subscale scores of the ASQ-3 in Nepali preschoolers

VARIABLES		N	AA			DHA		
Linear regression			Coeff.	CI	*P*	Coeff.	CI	*P*
Total ASQ-3	crude	283	3.61	(− 0.28, 7.50)	0.07	8.48	(−2.30, 19.27)	0.12
	adjusted^a^	282	−0.56	(− 4.40, 3.29)	0.77	− 1.60	(− 11.39, 8.209	0.75
Logistic regression			OR					
Total ASQ-3	crude	283	0.56	(0.36, 0.87)	0.01	0.26	(0.08, 0.81)	0.02
	adjusted^a^	282	0.85	(0.52, 1.40)	0.52	0.65	(0.20, 2.13)	0.48
Subscales			OR					
Communication	crude	283	0.44	(0.23, 0.82)	0.01	0.10	(0.02, 0.51)	0.01
	adjusted^a^	282	0.72	(0.35, 1.46)	0.36	0.28	(0.04, 1.90)	0.19
Gross motor	crude	283	0.83	(0.46, 1.50)	0.54	0.39	(0.09, 1.73)	0.22
	adjusted^a^	282	1.11	(0.58, 2.12)	0.75	0.73	(0.13, 4.19)	0.72
Fine motor	crude	283	1.01	(0.68, 1.79)	0.70	1.33	(0.40, 4.45)	0.64
	adjusted^a^	282	1.40	(0.87, 2.26)	0.16	2.38	(0.71, 8.01)	0.16
Problem Solving	crude	283	0.71	(0.42, 1.21)	0.21	0.49	(0.10, 2.32)	0.37
	adjusted^a^	282	1.01	(0.60, 1.72)	0.96	1.03	(0.24, 4.37)	0.97
Personal Social	crude	283	1.07	0.52, 2.23)	0.85	1.62	(0.28, 9.40)	0.59
	adjusted^a^	282	1.34	(0.64, 2.78)	0.44	2.78	(0.45, 17.14)	0.27

Linear and logistic regression models adjusted for clustering

^a^Adjusted for sex, age at baseline and weight-for-age z-scores at baseline and age at testing

OR > 1 indicates an increased risk of being in the lowest 25th percentile for each increment of one biomarker units

Associations between DHA and AA and the NEPSY II subtests in the Nepalese preschoolers are presented in Table 7. We found no notable associations between the NEPSY II subtests with either AA or DHA in the linear regression models.

**Table 7:** Crude and adjusted^b^ associations between AA and DHA and the NEPSY-II subtests in Nepali preschoolers^a^

VARIABLES			AA			DHA		
		N	Coefficient	CI	*P*	Coefficient	CI	*P*
Attention and Executive Functioning								
Inhibition-Naming Completion Time Total - raw	crude	282	−3.34	(−7.91, 1.23)	0.15	−2.90	(−14.18, 8.38)	0.61
	adjusted	281	−3.26	(−7.44, 0.93)	0.12	−1.95	(− 12.85, 8.96)	0.72
Inhibition-Inhibition Completion Time Total- raw	crude	282	−6.00	(−12.01, 0.02)	0.03	−10.60	(−27.63, 6.44)	0.22
	adjusted	281	−6.84	(−13.59, − 0.09)	0.05	− 11.99	(− 30.27, 6.29)	0.19
Inhibition Total Errors - raw	crude	282	−0.41	(−3.33, 2.52)	0.78	1.96	(−4.55, 8.48)	0.55
	adjusted	281	0.19	(−2.49, 2.86)	0.89	3.43	(−2.88, 9.74)	0.28
Statue Total Score - raw	crude	282	−0.11	(−0.56, 0.34)	0.64	0.00	(−1.31, 1.31)	1.00
	adjusted	281	−0.13	(− 0.61, 0.35)	0.60	− 0.03	(−1.40, 1.33)	0.96
Sensorimotor								
Visuo-motor Precision Total Completion Time - raw	crude	282	4.69	(−6.23, 15.62)	0.39	4.69	(−22.81, 32.18)	0.73
	adjusted	281	3.69	(−6.73, 14.11)	0.48	3.38	(−22.71, 29.47)	0.80
Social Perception								
Affect Recognition Total Score - raw	crude	282	0.33	(−0.46, 1.14)	0.40	0.93	(−1.22, 3.07)	0.39
	adjusted	281	0.20	(−0.56, 0.95)	0.61	0.69	(−1.34, 2.72)	0.50
Visuospatial Processing								
Geometric Puzzles - raw	crude	282	0.63	(0.06, 1.21)	0.03	1.80	(0.22, 3.40)	0.03
	adjusted	281	0.60	(0.03, 1.16)	0.04	1.70	(0.07, 3.32)	0.04
Block Construction - raw	crude	282	0.24	(−0.05, 0.53)	0.10	0.31	(−0.50, 1.13)	0.44
	adjusted	281	0.21	(−0.06, 0.49)	0.12	0.22	(−0.60, 1.03)	0.60

^a^All models are adjusted for clustering

^b^Adjusted for sex, age at baseline and weight-for-age z score at baseline and age at testing

## Discussion

Overall, we did not find any associations between early DHA and AA status and developmental status by ASQ-3 and cognitive functioning by NEPSY II five years later in a sample of 303 children from Nepal.

The lack of associations between early DHA and AA status and neurodevelopmental scores in our study is in accordance with literature that report no beneficial effect of n-3 LCPUFA supplementation on cognitive function in full-term infants [14, 21, 22, 36–39]. However, four recent reviews reported that n-3 LCPUFA supplementation in term infants might have a potential effect on later child development [2, 16, 18, 23]. Few studies have in general investigated the long-term associations between early fatty acid status and cognitive function. A longitudinal examination of the relationship over time may be needed given that the subtle effects of fatty acids on the brain may not show up until later in childhood [3]. Two studies have reported beneficial effects on some specific aspects of cognition (attention, speed of processing, and problem solving), suggesting that the effects of LCPUFA supplementation in early life may be better detectable at a later age for specific cognitive functions [40, 41]. Some other studies assessed cognitive development of children between 6 or 9 years of age and found no overall positive effect of fatty acids on general cognitive functioning [42–44].

According to Carlson, the effect of DHA and AA status and supplementation in infancy has been largely evaluated through global developmental assessments focused on attainment of normative milestones, although more granular measures of specific cognitive function may be more sensitive markers of the effects of LCPUFA supplementation [19]. Researchers now have access to techniques to measure brain electrical interconnectivity and brain structure and function; these techniques were not used in the early studies of DHA and AA supplementation and could explain the lack of an effect [23].

Trials have found a positive effect of n-3 LCPUFA supplementation on cognitive function in low birth weight (LBW) infants at risk of deficiency in certain fatty acids, including DHA [3, 4]. Regional estimates of LBW include 28% in South Asia [45]. In our study sample, 14% of the infants had LBW, however, we did not find any difference in either essential fatty acid concentration or the ASQ-3 and NEPSY II scores when the analyses was stratified on children less than 2500 g and children 2500 g and above.

From the literature, there are some evidence for a favorable effect of n-3 LCPUFA in children deficient in fatty acids [17, 18]. In many low to middle-income countries, including Nepal, diets are lacking EFA and low fatty acids status would be expected. Despite substantial variability in fatty status, we found better than expected concentrations of erythrocyte DHA and AA among breastfeeding women and their infants in Nepal [26], and this may explain the lack of associations between early DHA and AA status and developmental scores five years later in the current study. The main dietary source of fat among the Nepali women was sunflower, soybean, sesame, and mustard oils. The relatively high concentration of LA in the cooking oils ensured a substantial intake of this essential fatty acid in their diet. For ALA, the concentration in cooking oil was considerably lower, giving a much lower total intake of ALA. Cooking oils and ghee had very low concentrations of AA; no item provided more than 1 mg/g, and DHA was not detected in any of the analyzed food items. Some of the vegetable oils commonly used in food preparation contained EPA and may be a significant dietary source [27]. Analysis of differences in genetic polymorphisms (FADS) should be taken into consideration in future studies [23]. Further, child development may be influenced by several other nutritional inadequacies, such as undernutrition, iron- zinc and B-vitamin deficiency, in addition to inadequate childcare and home environment [1]. Our multivariable models were adjusted for potential confounders including age, nutritional status, exclusive breastfeeding, maternal education and family situation (Table 1), however, given the observational nature of our dataset we cannot rule out the possibility for unmeasured negative confounding that may have led to bias in our study.

Even though we did not find any association between DHA and AA status and the neurodevelopmental scores five years later in this follow-up study, it is well established that fatty acids are crucial for optimal brain development [1]. DHA and AA are major components of the brain, and play an important role in the structure and function of all cell membranes [6] through neuron proliferation, synapse formation, pruning, and myelination [1]. Why then is the crucial role of fatty acids in brain development not reflected in the numerous RCT and epidemiological studies aiming to link fatty acids and cognitive performance, and why do systematic reviews and meta-analyses often show conflicting results? Firstly, comparison of results from different studies is often compromised by differences in age of supplementation, different dosages and different lengths of supplementation, lack of consideration of FADS polymorphisms, the inclusion of populations only of a specific age, or exclusion of healthy individuals only. Secondly, the large heterogeneity in cognitive tests may complicate the comparison of the potential effect of fatty acid status on brain development [3, 16, 19, 23].

To the best of our knowledge, this is one of the first studies on fatty acid status and child development in South Asia and the first from Nepal. In addition to a representative sample of children, one of the strengths of our study is that fatty acid status of the infants was measured in the infants’ red blood cells, as recommended [18], not maternal fat intake or maternal fatty acid status during pregnancy and lactation. A trained local psychologist performed the neurodevelopmental assessment by the ASQ-3 and NEPSY II; however, it should be noted, that these tools have not been validated for a Nepalese setting. The assessment has been used previously in similar settings and the psychometric properties are comparable to those of other studies [33]. The fact that 160 of the children were above the recommended age range for the ASQ-3 assessment may have compromised our results and could explain the null findings in the models with the ASQ-3 scores. In spite of the limitation that approximately half of the children where above the recommended age range for the ASQ-3, the data has previously yielded findings in a study on vitamin B-12 status and child development, which give support to the validity of the developmental variables [28].

In conclusion, no notable associations between early DHA and AA status and overall developmental status and cognitive functioning five years later, were found in this study among children from a peri-urban area of Nepal. It should be noted that better than expected concentrations of erythrocyte DHA and AA were found, and the ASQ scores were left-skewed, which limited the ability to identify associations.

## Abbreviations

AA: arachidonic acid
ALA: a-linolenic acid
ASQ-3: Ages and Stages Questionnaire 3rd. ed.
CNS: central nervous system
DHA: docosahexaenoic acid
HAZ: height-for-age
LA: linoleic acid
LBW: low birth weight
LCPUFA: long chain polyunsaturated fatty acids
RBC: red blood cells
WAZ: weight-for-age
